# Supplementary material for: Deletion or immunotherapeutic blockade of FcγRIIb (CD32b) impairs α-Syn propagation in vivo
Source: Acta Neuropathol. 2026 Sep 25;152(1):40. doi: 10.1007/s00401-026-03082-7 (PMC13615098; doi:10.1007/s00401-026-03082-7)
Supplement: Supplementary file 1 [file 401_2026_3082_MOESM1_ESM.pdf]

Supplementary Information

Table S1: List of Antibodies and Oligonucleotides

REAGENT	SOURCE	IDENTIFIER
**Antibodies (ICC)**		
Mouse anti-pSer129-α-Syn (81A)	BioLegend	825701
**Antibodies (IHC)**		
Rat anti-mFcγRII (AT130-5)	In House	AT130-5
Rabbit anti-hFcγRIIb (EP888Y)	Abcam	ab45143
Rabbit anti-mouse α-Syn	Abcam	ab212184
Rabbit anti-pSer129-α-Syn (EP1536Y)	Abcam	ab51253
Rabbit anti-Iba1	Conalab	CUK2222
Rat anti-CD64 (AT152-9)	Bio-Rad	MCA5997
Rabbit anti-TH	Abcam	ab41528
Biotinylated goat anti-mouse	Vector Laboratories	BA-9200
Biotin goat anti-rat	Vector Laboratories	BA-9400
Biotin goat anti-rabbit	Vector Laboratories	BA-1000
Alexa 568 goat anti-mouse	Invitrogen	A11004
Alexa 568 goat anti-rabbit	Invitrogen	A11011
Alexa 488 goat anti-rabbit	Invitrogen	A11008
Alexa 568 goat anti-rat	Invitrogen	A11077
**Oligonucleotides**		
Gapdh Forward	Sigma	5’-TGAACGGGAAGCTCACTGG
Gapdh Reverse	Sigma	5’-TCCACCACCCTGTTGCTGTA
Snca Forward	Sigma	5’-GGGAGAGGCGCTGACAAATC
Snca Reverse	Sigma	5’-GTATTTTTGCTCCACACGGCTC
Fcgr1 Forward	Sigma	5’-TACTTTGGGTTCCAGTCGGT
Fcgr1 Reverse	Sigma	5’-CCTGTATTCGCCACTGTCCT
Fcgr2b Forward	Sigma	5’-AACCATCACGCTAAGGTGCC
Fcgr2b Reverse	Sigma	5’-TGCGACAGCAATCCCAGTGA
FCGR2B Forward	Sigma	5’-GGTCACTGGGATTGCTGTAGCGG
FCGR2B Reverse	Sigma	5’-CCAGAGCATCCGGGTGCAT
Fcgr3 Forward	Sigma	5’-TGCTTTTGCAGACAGGCAGAGT
Fcgr3 Reverse	Sigma	5’-GCAGCAGCCAGTCAGAAATCAC
Fcgr4 Forward	Sigma	5’-GAAAGCCTCATCCCACACCA
Fcgr4 Reverse	Sigma	5’-CTGAAGCAATAGCCAGCCCATA

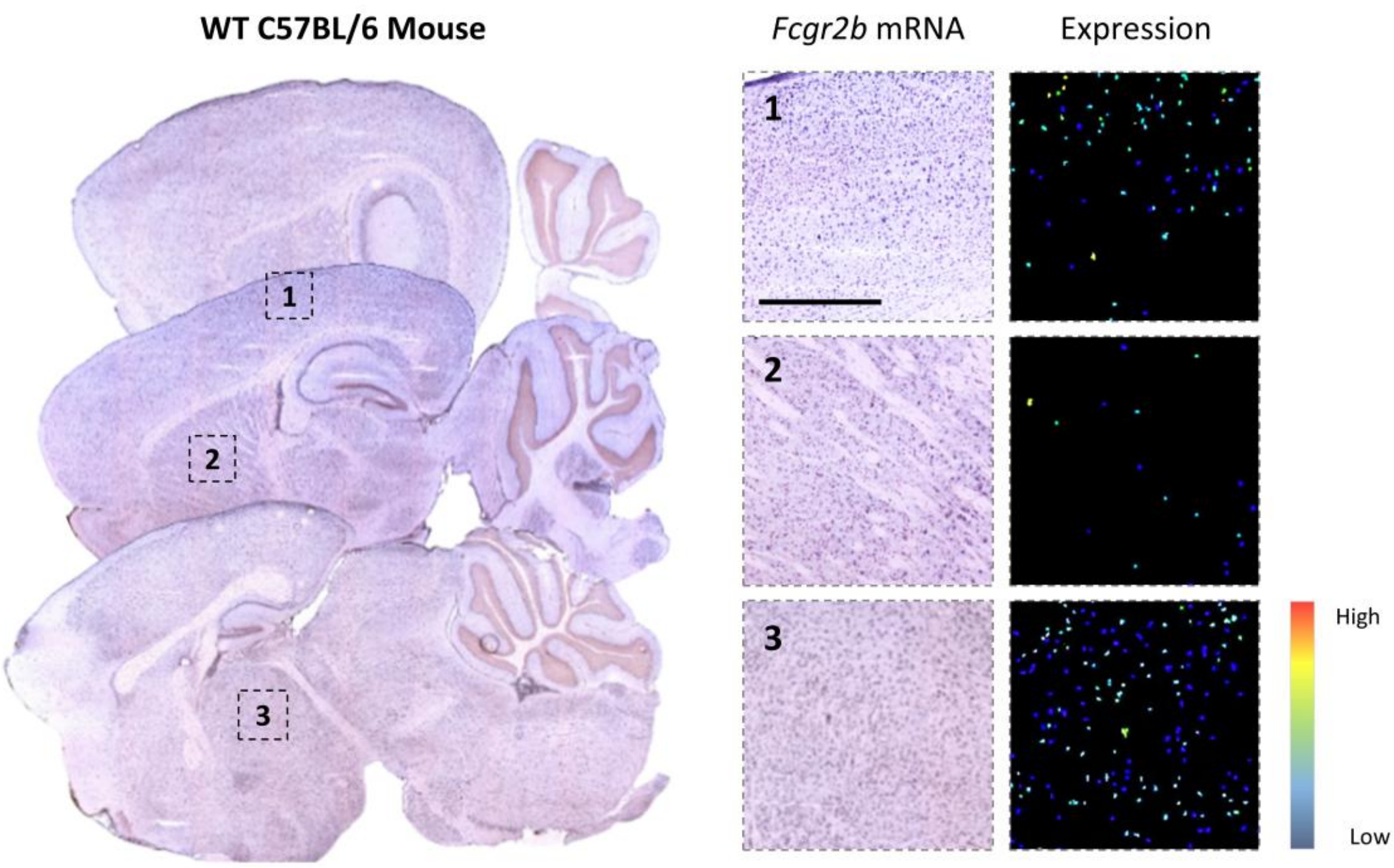

Fig. S1. Expression pattern of *Fcgr2b* in the WT mouse brain from the Allen Mouse Brain Atlas. *In-situ* hybridisation and corresponding expression heat map illustrating *Fcgr2b* mRNA distribution in sagittal sections of the adult WT C57BL/6 mouse brain (postnatal day 56). The probe targeted exons 1-7 of the *Fcgr2b* transcript. Heat-map rendering highlights regional differences in expression, with enlarged panels depicting representative areas of interest. Scale bar = 500 µm. Data source: Allen Brain Atlas, © 2015 Allen Institute for Brain Science (https://mouse.brain-map.org/).

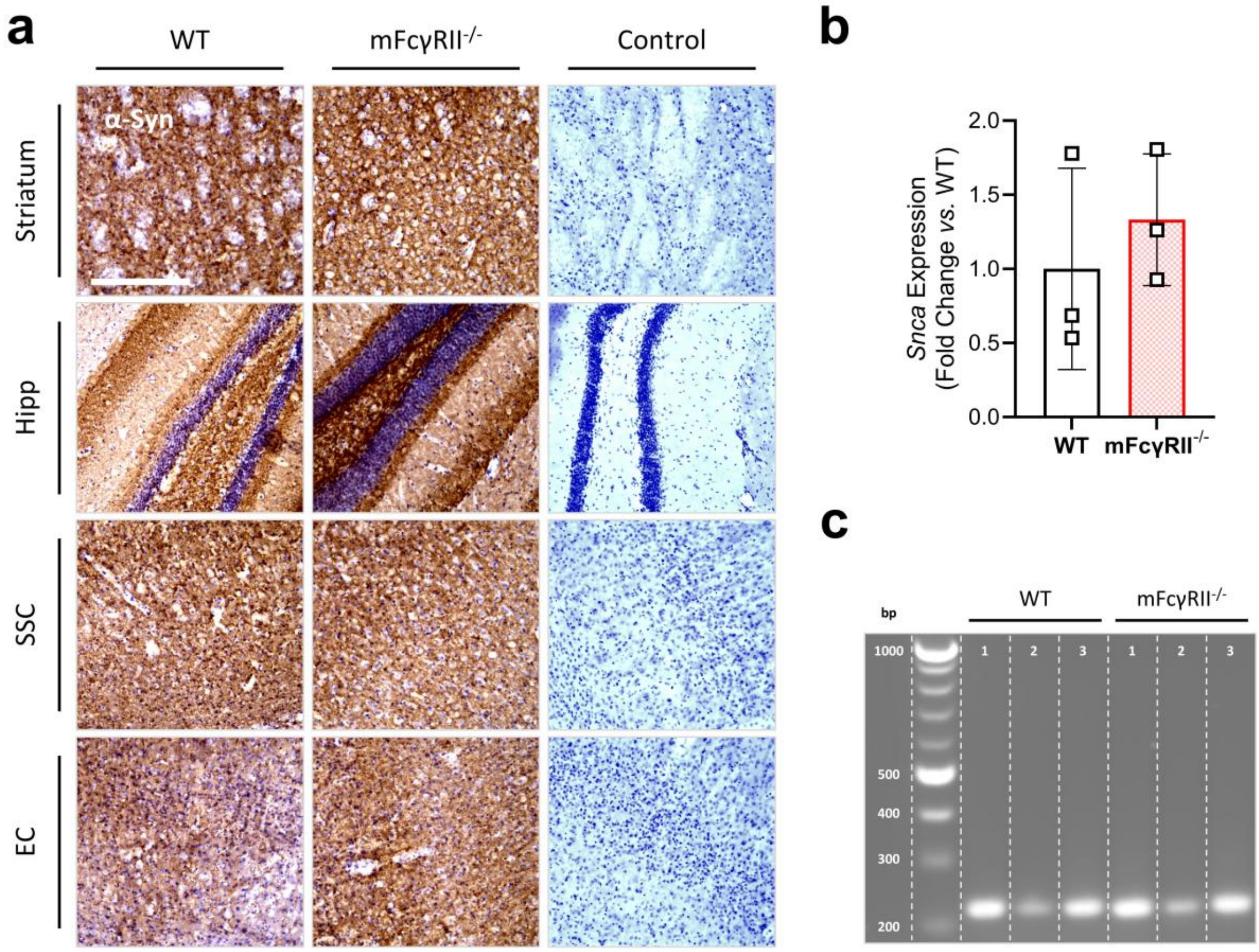

Fig. S2. α-Syn expression in brain tissue from WT and mFcγRII$^{-/-}$ mice. (**a**) Representative images of immunohistochemical total mouse α-Syn staining in the contralateral striatum, hippocampus (Hipp), somatosensory cortex (SSC) and entorhinal cortex (EC) of WT and mFcγRII$^{-/-}$ mice at 90 dpi PFF α-Syn. Control panels indicate tissue devoid of primary antibody. Scale bar = 100 µm. (**b**) *Snca* mRNA expression in brain homogenates from WT and mFcγRII$^{-/-}$ mice measured by RT-qPCR and expressed as fold change relative to the mean of WT (*n* = 3 mice per genotype). (**c**) Representative agarose gel of *Snca* RT-PCR products from naïve WT and mFcγRII$^{-/-}$ mice (*n* = 3 mice per genotype).

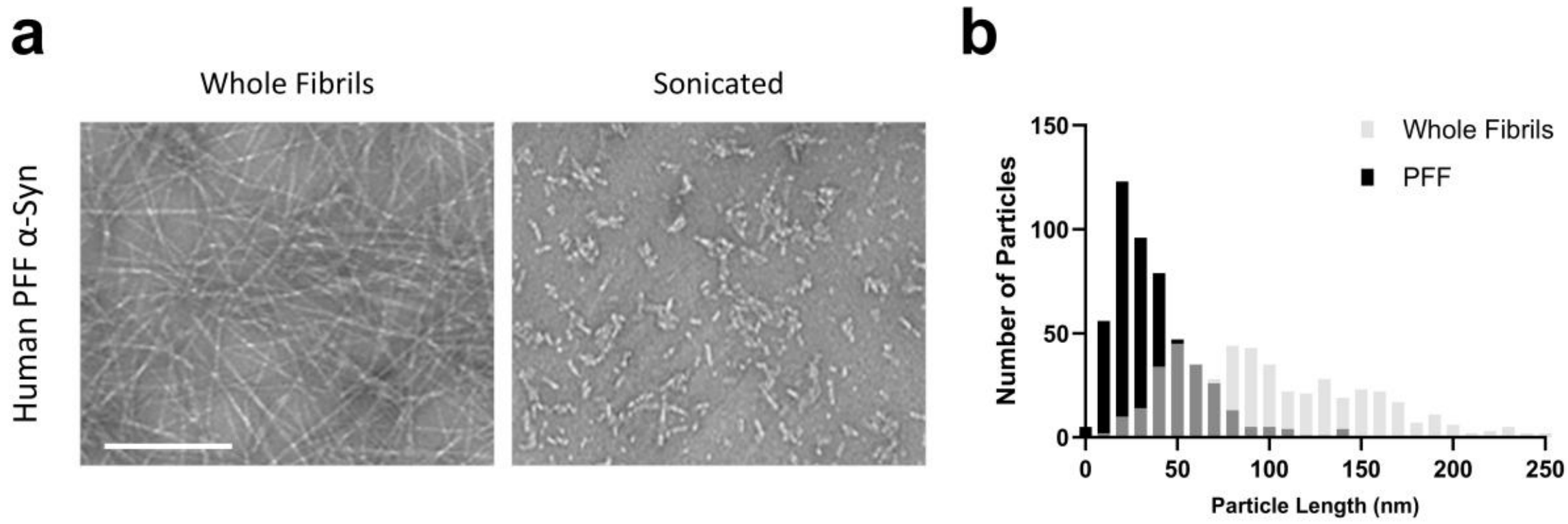

Fig. S3. Preparation of recombinant human PFF α-Syn. (**a**) Representative transmission electron microscopy images of whole human PFF α-Syn fibrils and sonicated PFF α-Syn seeds. Scale bar = 200 nm. (**b**) Graph showing the size distribution of whole fibrils (grey) and PFF seeds (black) depicted as length in nm. Mean length of PFF α-Syn = 34.3 nm ± 2.37 (*n* = 100).

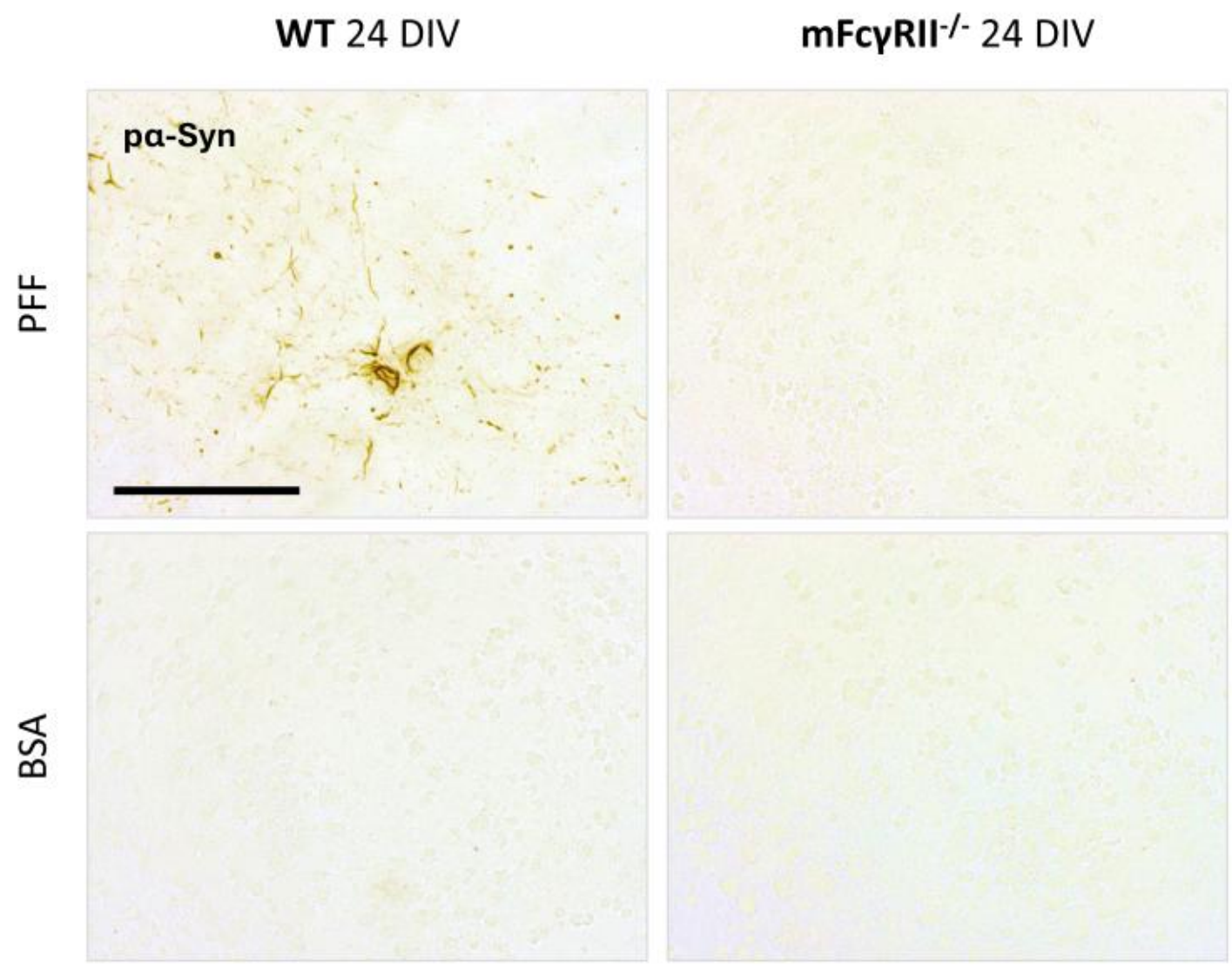

Fig. S4. pα-Syn^{+} staining is exclusive to PFF-treated WT primary neuronal cultures. Representative images of immunocytochemical pα-Syn staining in primary neurons derived from WT and mFcγRII$^{-/-}$ embryos at 24 days *in-vitro* (DIV) following exposure to PFF α-Syn or bovine serum albumin (BSA) control at DIV 10. Scale bar = 100 μm.

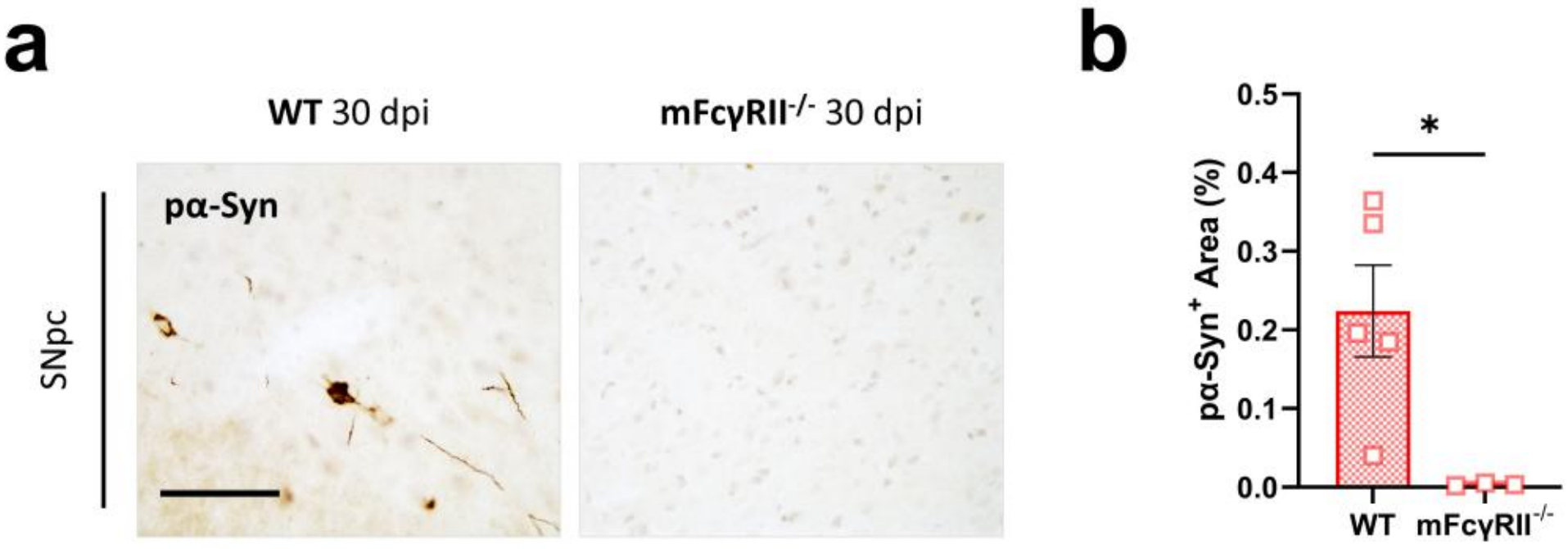

Fig. S5. Quantification of pilot pα-Syn+ pathology in WT and mFcγRII$^{-/-}$ mice at 30 dpi. (**a**) Representative images of immunohistochemical pα-Syn staining in the ipsilateral Substantia Nigra *pars compacta* (SNpc) of WT or mFcγRII$^{-/-}$ mice injected with PFF α-Syn at 30 dpi. Scale bar = 50 µm. (**b**) Quantification of pα-Syn+ immunoreactivity (% area) in the SNpc for each genotype. Data are shown as mean ± SEM with all individual data points. WT PFF; *n* = 5, mFcγRII$^{-/-}$ PFF; *n* = 3 animals. Statistical significance was determined by t-test with Welch's correction (*p < 0.05). Data were generated as part of an independent pilot study to determine initial effect size of mFcγRII$^{-/-}$ in the PFF model and to inform cohort sizes used in principal experiments.

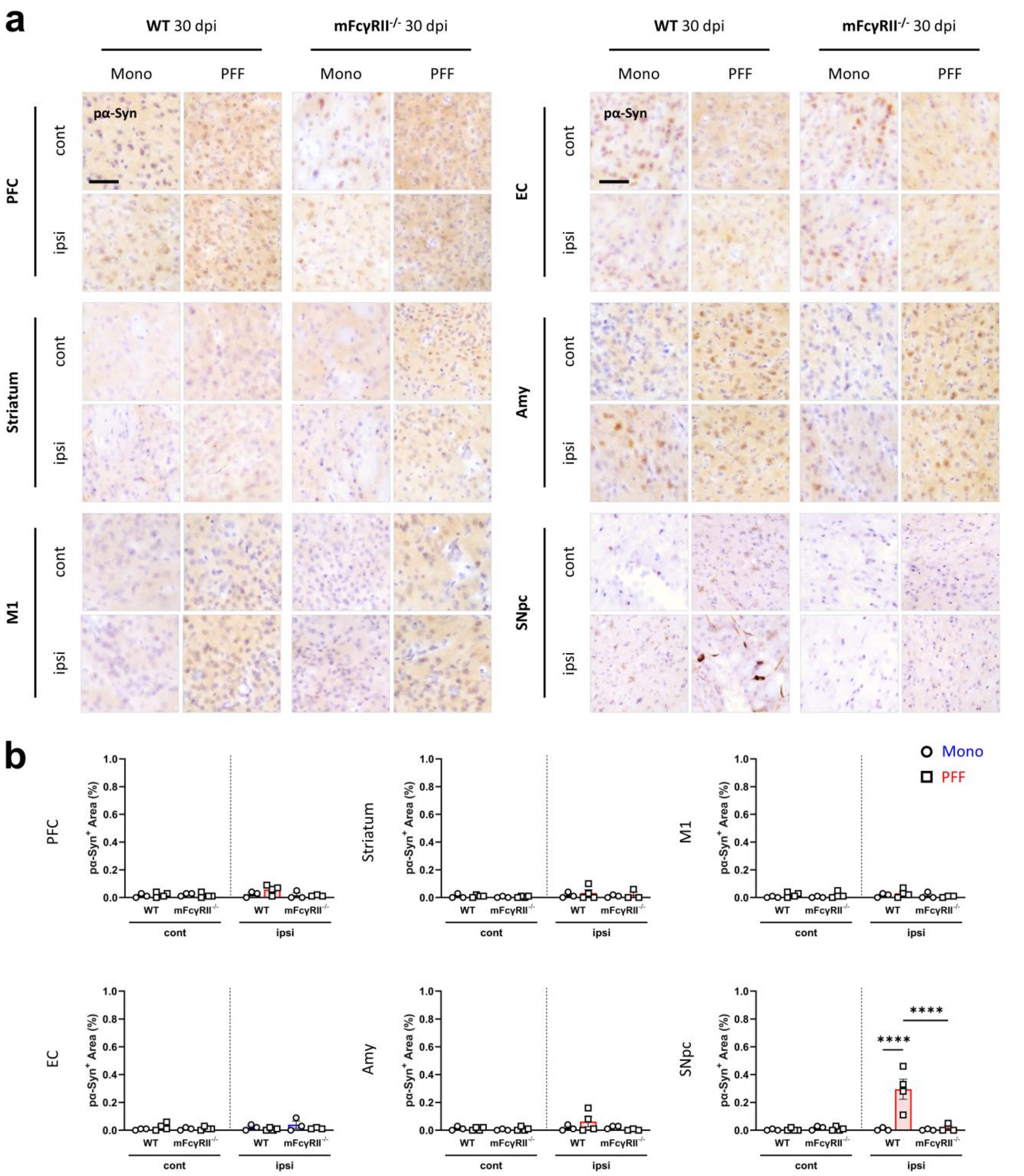

Fig. S6. pα-Syn accumulation in WT and mFcγRII$^{-/-}$ mice at 30 dpi. (**a**) Representative images of immunohistochemical pα-Syn staining in contralateral (cont) and ipsilateral (ipsi) hemispheres of WT or mFcγRII$^{-/-}$ mice injected with PFF or Mono α-Syn at 30 dpi. PFC; prefrontal cortex, M1; primary motor cortex, EC; entorhinal cortex, Amy; amygdala, SNpc; Substantia Nigra *pars compacta*. Sections were counterstained with haematoxylin. Scale bar = 50 μm. (**b**) Quantification of pα-Syn^{+} immunoreactivity (% area) at 30 dpi in indicated brain regions and groups from (a). WT Mono; *n* = 3, mFcγRII$^{-/-}$ Mono; *n* = 3, WT PFF; *n* = 4, mFcγRII$^{-/-}$ PFF; *n* = 3 animals. Data are mean ± SEM. Statistical significance was determined using two-way ANOVA with Tukey's multiple comparisons test (*****p*<0.0001).

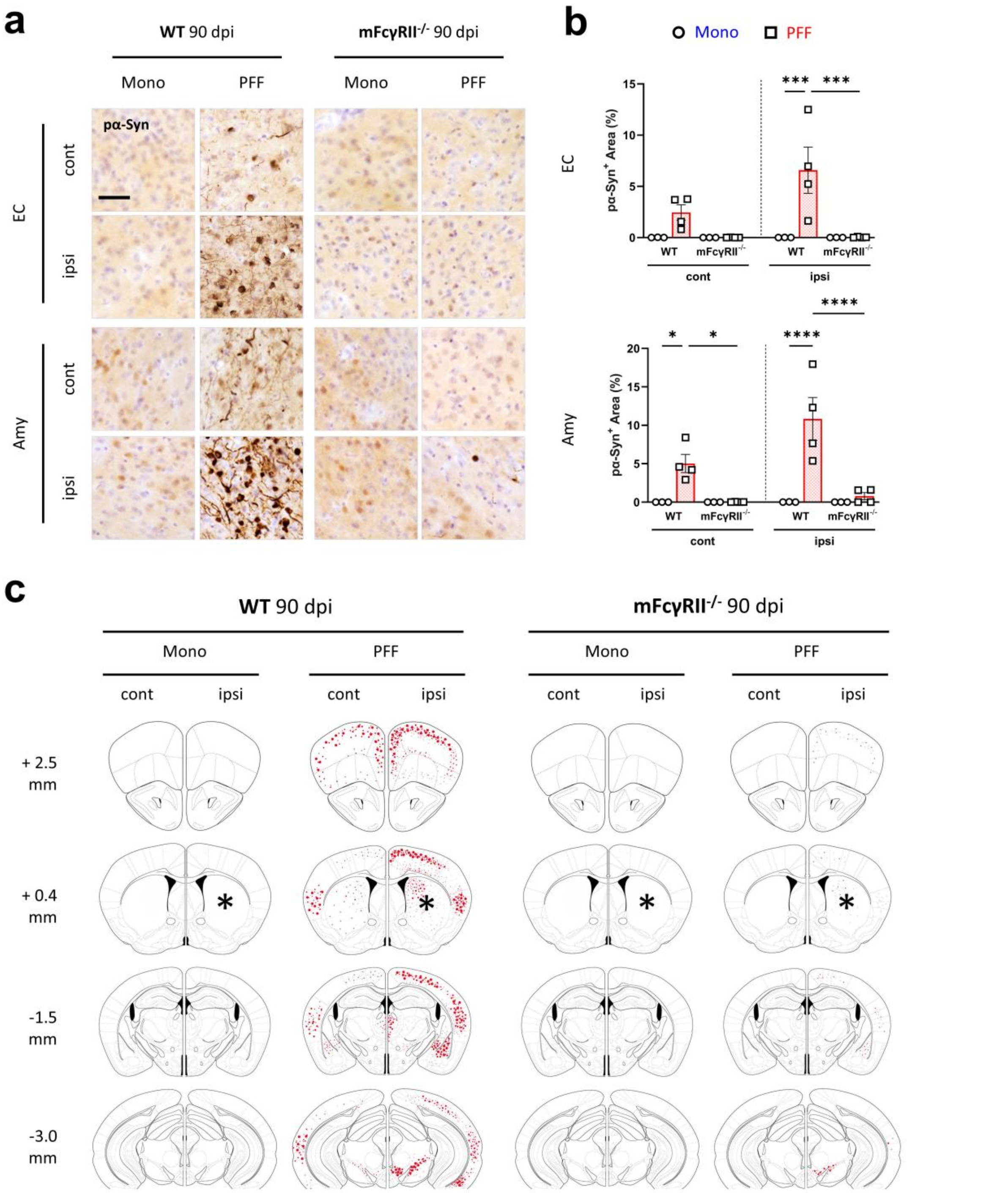

Fig. S7. Regional distribution of pα-Syn in WT and mFcγRII$^{-/-}$ mice at 90 dpi (additional brain regions). (**a**) Representative images of immunohistochemical pα-Syn staining in contralateral (cont) and ipsilateral (ipsi) hemispheres of WT or mFcγRII$^{-/-}$ mice injected with PFF or Mono α-Syn at 90 dpi. EC; entorhinal cortex, Amy; amygdala. Sections were counterstained with haematoxylin. Scale bar = 50 μm. (**b**) Quantification of pα-Syn^{+} immunoreactivity (% area) at 90 dpi in indicated brain regions and groups from (**a**). WT Mono; *n* = 3, mFcγRII$^{-/-}$ Mono; *n* = 3, WT PFF; *n* = 4, mFcγRII$^{-/-}$ PFF; *n* = 4 animals. Data are mean ± SEM. Statistical significance was determined using two-way ANOVA with Tukey's multiple comparisons test (**p*<0.05, ****p*<0.001, *****p*<0.0001). (**c**) Distribution map of pα-Syn^{+} immunoreactivity (represented as red dots) across the whole brain at 90 dpi. Experimental groups are indicated alongside both contralateral (cont) and ipsilateral (ipsi) hemispheres. Asterisks denote the injection site (ipsi striatum), and adjacent numbers represent distance from Bregma in the anterior-posterior plane in mm.

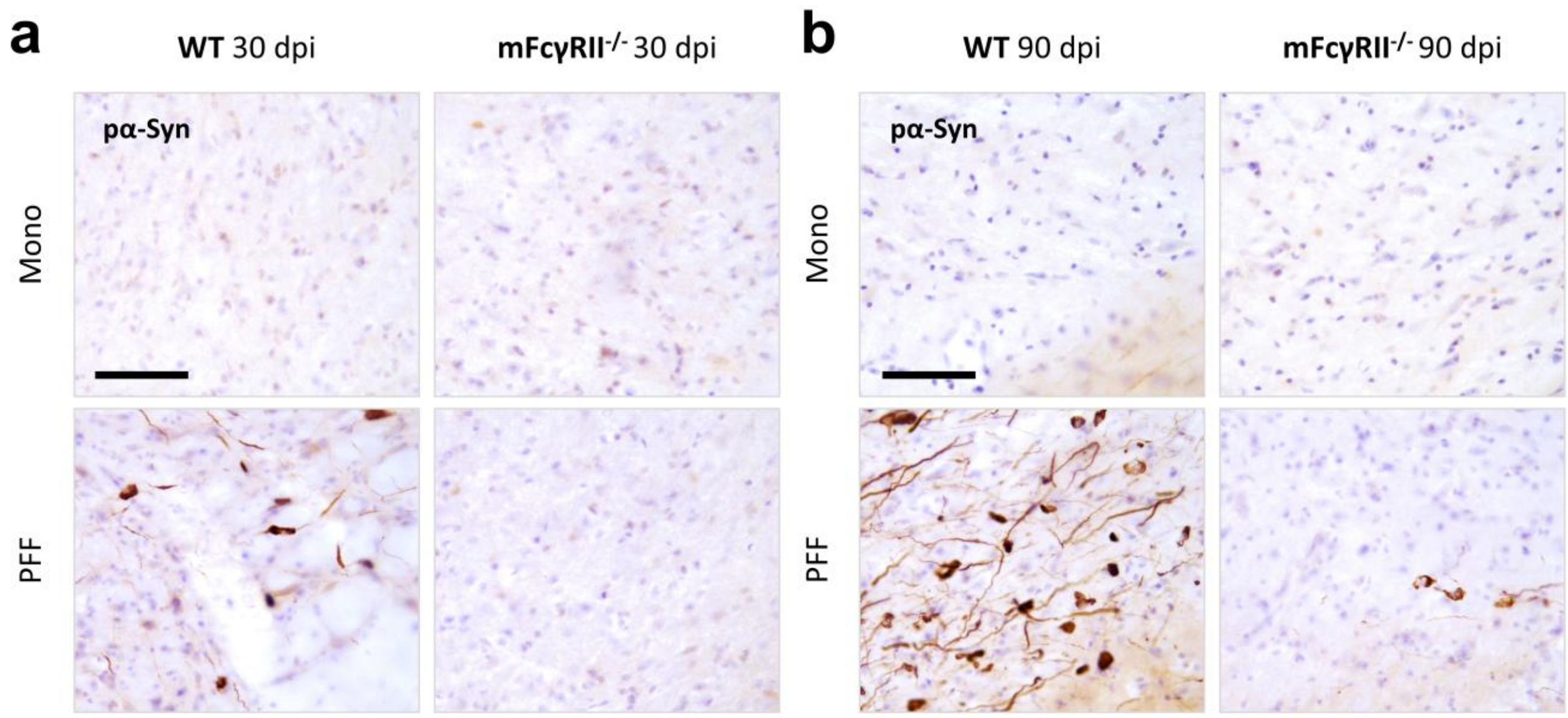

Fig. S8. Enlarged images of pα-Syn pathology in WT and mFcγRII$^{-/-}$ mice. Representative images of immunohistochemical pα-Syn staining in the ipsilateral Substantia Nigra *pars compacta* (SNpc) of WT and mFcγRII$^{-/-}$ mice at (**a**) 30 dpi and (**b**) 90 dpi following unilateral intrastriatal injection of Mono or PFF α-Syn. Scale bar = 50 µm.

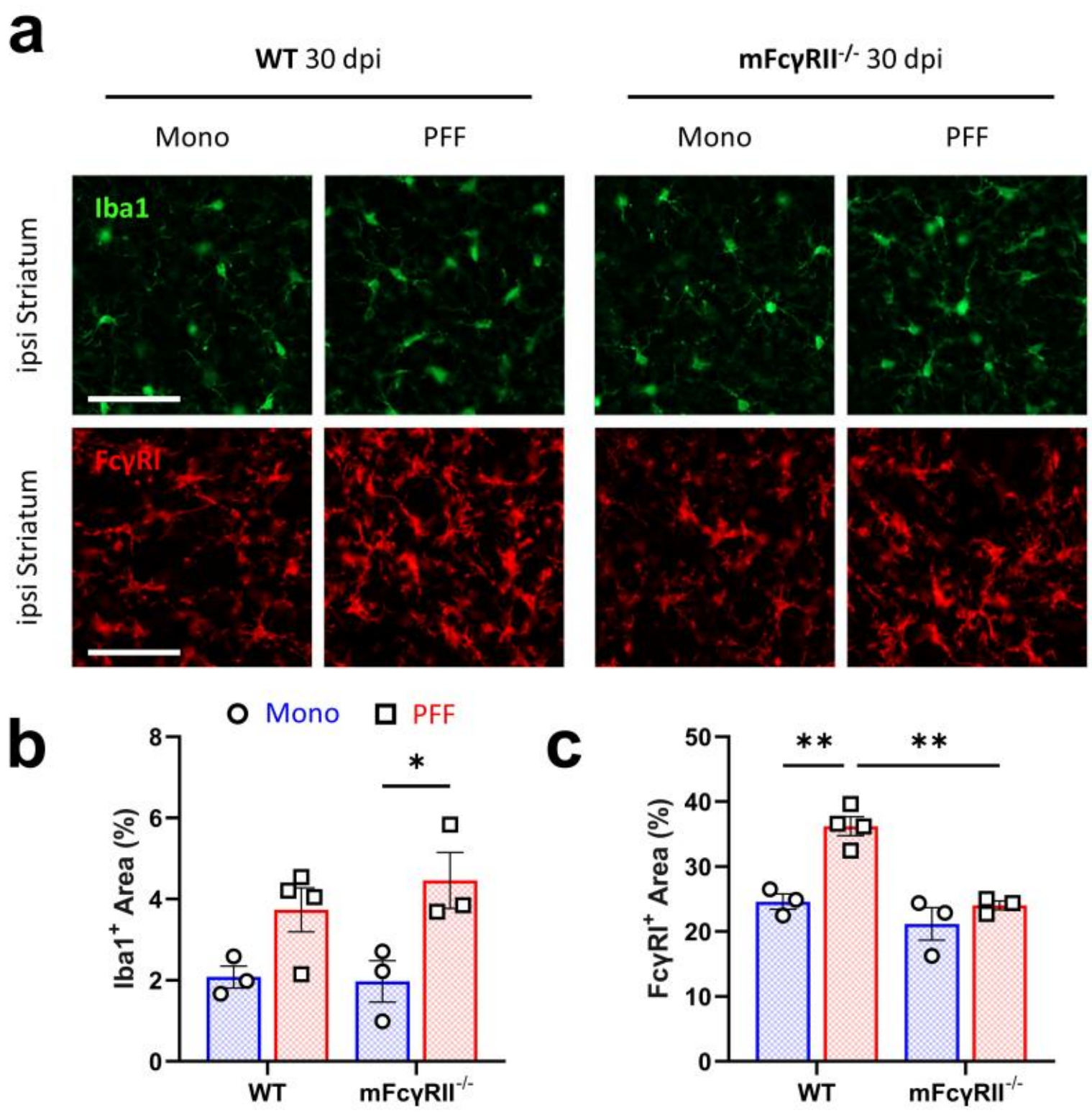

Fig. S9. Microglial reactivity in the striatum of WT and mFcγRII$^{-/-}$ mice at 30 dpi. (**a**) Representative images of immunofluorescent Iba1 (green) and FcγRI (red) in the ipsilateral (ipsi) striatum of WT and mFcγRII$^{-/-}$ mice injected with PFF or Mono α-Syn at 30 dpi. Scale bars = 50 µm. (**b, c**) Quantification of (**b**) Iba1^{+} and (**c**) FcγRI^{+} immunoreactivity (% area) at 30 dpi in the ipsi striatum. WT Mono; *n* = 3, mFcγRII$^{-/-}$ Mono; *n* = 3, WT PFF; *n* = 4, mFcγRII$^{-/-}$ PFF; *n* = 3 animals. Data are mean ± SEM. Statistical significance was determined using two-way ANOVA with Tukey's multiple comparisons test (**p*<0.05, ***p*<0.01).

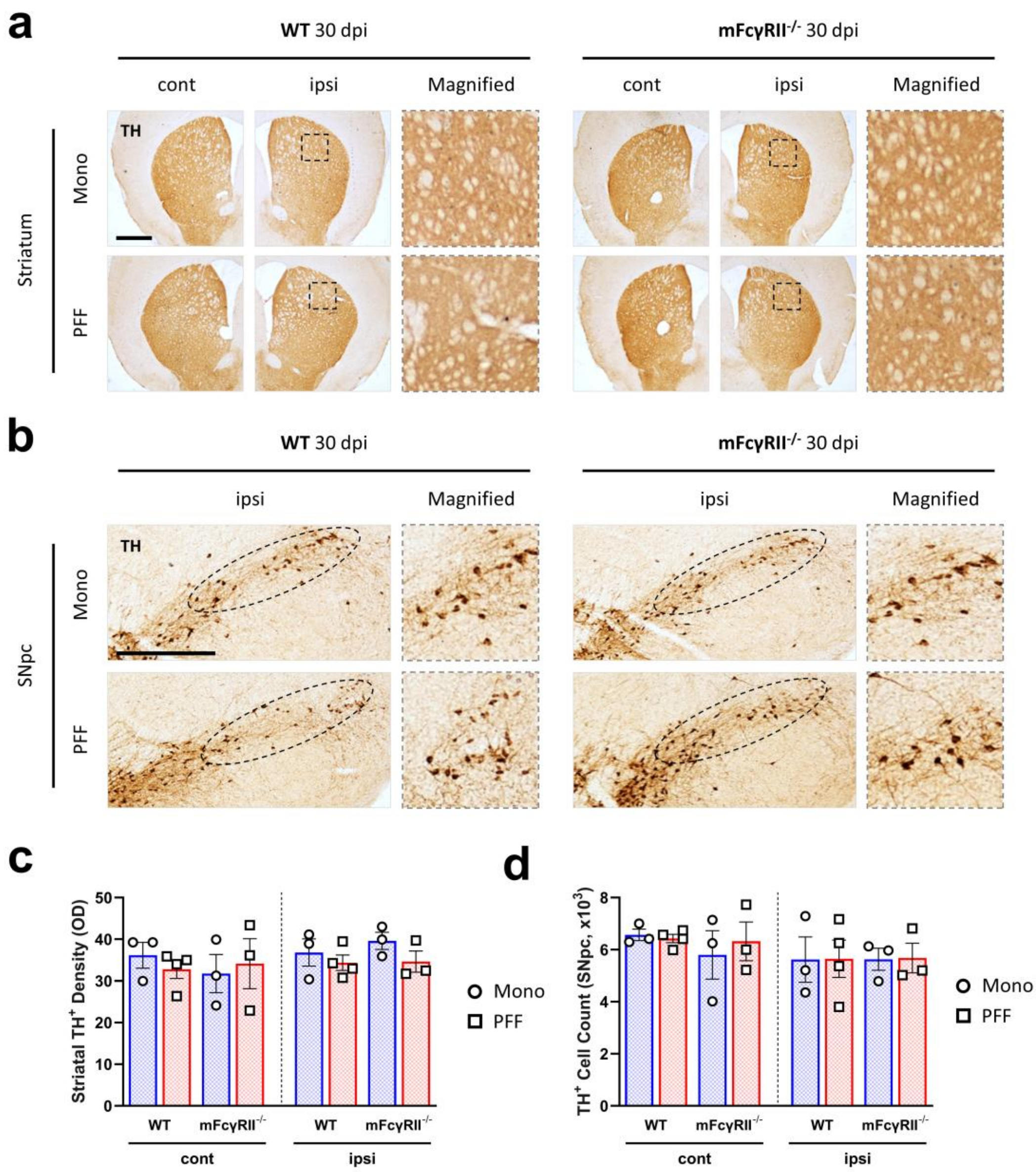

Fig. S10. TH expression in WT and mFcγRII$^{-/-}$ mice at 30 dpi. (**a, b**) Representative images of immunohistochemical TH staining in (**a**) the contralateral (cont) and ipsilateral (ipsi) striatum and (**b**) the ipsi Substantia Nigra *pars compacta* (SNpc) of WT and mFcγRII$^{-/-}$ mice injected with PFF or Mono α-Syn at 30 dpi. Magnified images are shown adjacent to each panel. Scale bars = 1 mm (a) and 200 µm (**b**). (**c**) Quantification of TH^{+} optical density (OD) in the dorsal striatum and (**d**) TH^{+} cells in the SNpc. WT Mono; *n* = 3, mFcγRII$^{-/-}$ Mono; *n* = 3, WT PFF; *n* = 4, mFcγRII$^{-/-}$ PFF; *n* = 3 animals. Data are represented as mean ± SEM.

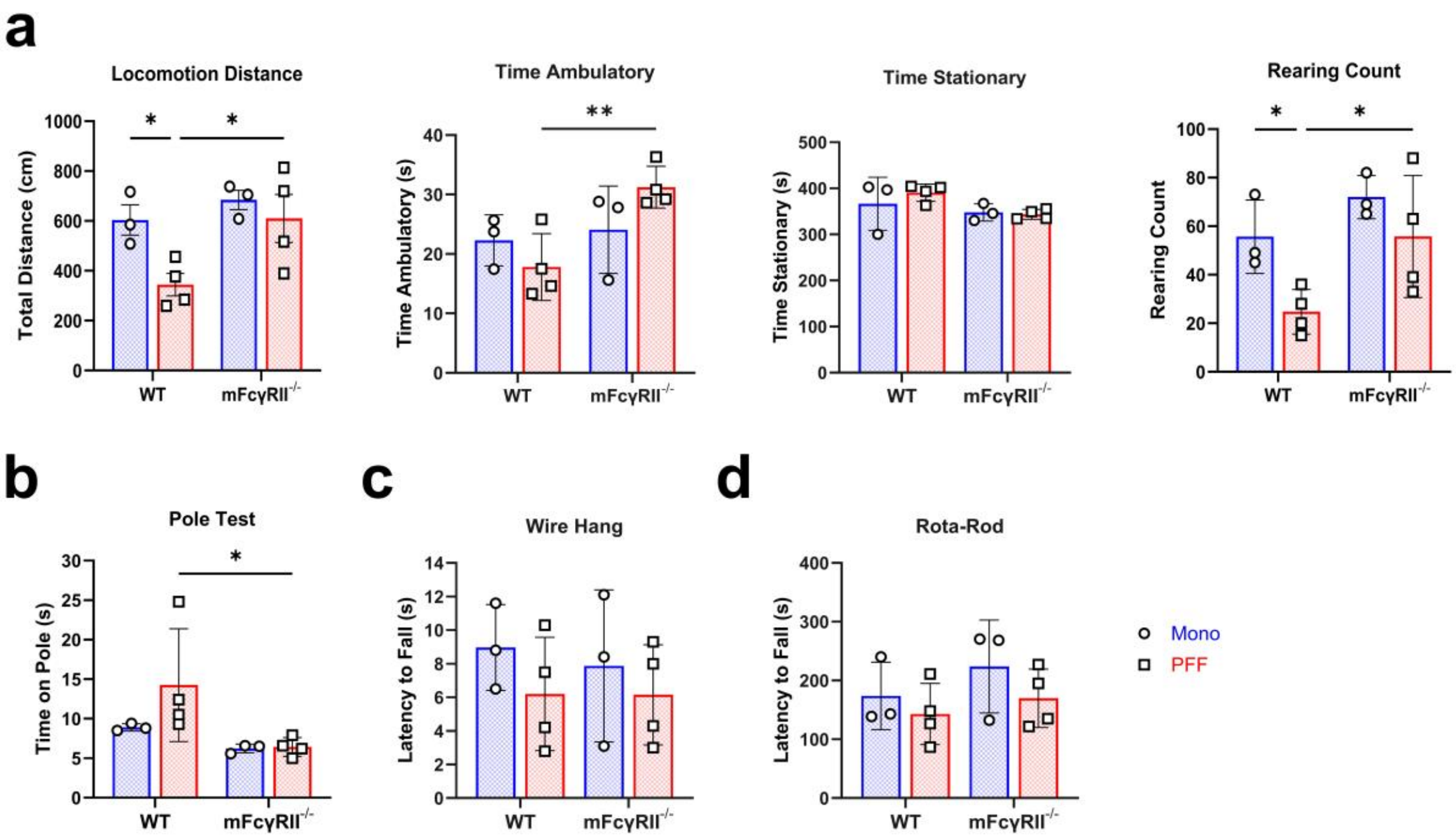

Fig. S11. Behavioural assessments in WT and mFcγRII$^{-/-}$ mice at 90 dpi. (**a**) Total distance travelled, time ambulatory, time stationary, and rearing count in the open-field test at 90 dpi. (**b**) Total time spent descending the pole in the pole test at 90 dpi. Each point represents the mean of 3 independent tests per mouse. (**c**) Wire hang test assessing neuromuscular strength and coordination. Each point represents the latency to fall from the apparatus averaged over 3 trials per animal. (**d**) Rota-rod test evaluating motor performance and balance. Each point represents the latency to fall from the apparatus averaged over 3 trials per animal. WT Mono, *n* = 3; mFcγRII$^{-/-}$ Mono, *n* = 3; WT PFF, *n* = 4; mFcγRII$^{-/-}$ PFF, *n* = 4 animals. Data are mean ± SD. Statistical significance was determined using two-way ANOVA with Tukey's multiple comparisons test (*p<0.05, **p<0.01).
